# Photocatalytic formation of a gas permeable layer selectively deposited on supported metal nanoparticles for sintering-resistant thermal catalysis†

**DOI:** 10.1039/d2na00703g

**Published:** 2022-12-19

**Authors:** Ayato Takabayashi, Fuminao Kishimoto, Hiroto Tsuchiya, Hitoshi Mikami, Kazuhiro Takanabe

**Affiliations:** a Department of Chemical System Engineering, School of Engineering, The University of Tokyo 7-3-1 Hongo Bunkyo-ku Tokyo 113-8656 Japan takanabe@chemsys.t.u-tokyo.ac.jp; b Honda R&D Co., Ltd. 4630 Shimotakanezawa Haga-machi, Hagagun Tochigi 321-3393 Japan

## Abstract

Nanoparticle aggregation of supported metal catalysts at high temperatures is a serious problem that causes a drop in catalytic performance. This study investigates the protection of metal nanoparticles from sintering by selectively forming nanoscale SiO_2_ shells on Pd supported on TiO_2_ by ultraviolet (UV) light irradiation. The proton-coupled reduction reaction increases the local pH around Pd nanoparticles, resulting in hydrolysis of tetraethoxyorthosilicate (TEOS) in only the vicinity of the metal. An apparent quantum efficiency of only 0.6% is obtained for the Pd/TiO_2_ catalyst in H_2_ evolution from ethanol-containing water under 370 nm excitation light. Therefore, the pH of raw slurry solution should be precisely controlled to that slightly below the threshold value for the TEOS hydrolysis reaction before the photodeposition. Transmission electron microscopy (TEM) and energy dispersive X-ray spectroscopy (EDX) clearly show that the particle size of the Pd nanoparticles (∼40 nm) with the SiO_2_ shell (∼20 nm) was almost unchanged by the high-temperature treatment at 900 °C in air, suggesting that the SiO_2_ shell prevented thermal aggregation of Pd nanoparticles. The Pd/TiO_2_ without SiO_2_ shell decoration exhibited a drop in the number of active sites, which was likely due to aggregation of the Pd catalysts. However, the number of active sites on the Pd@SiO_2_/TiO_2_ catalyst was maintained even after the catalyst was calcined at 900 °C. Consequently, the Pd@SiO_2_/TiO_2_ catalyst maintained its catalytic performance for simulated exhaust gas purification even after treatment at 900 °C. This study presents a methodology to produce sintering-tolerant supported metal nanoparticles using the photocatalytic gas permeable layer fabrication method.

## Abstract 2

This paper presents a new methodology to produce a sintering-resistant exhaust gas purification catalyst with ultrathin SiO_2_ decoration on already supported Pd metal nanoparticles using a vacuum-free photocatalytic layer deposition method.

## Graphical abstract

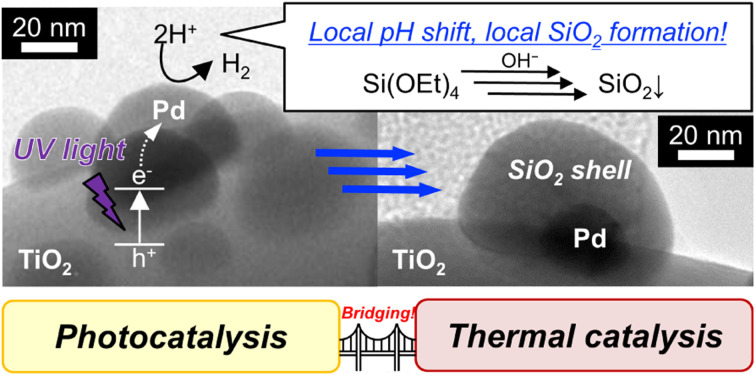

## Introduction

An exhaust-gas purification system for automobiles uses an excessive amount of noble metals due to aggregation of the supported metal catalysts under harsh conditions, such as high-temperature hydrothermal conditions.^1,2^ Aggregation is a phenomenon in which highly dispersed nanoparticles fuse on support materials at high temperature, resulting in particles with a large diameter and low dispersion.^3,4^ The aggregation is typically induced by increased diffusion coefficients due to temperature beyond the Tammann temperature,^5^ nanosizing of particles^6^ (Ostwald ripening^7^), or direct particle migration and coalescence.^8^ Suppressing the sintering could allow for high reactivity to be maintained for the exhaust-gas purification reaction, which could minimize the amount of noble metals (Rh, Pd, and Pt) used in the system, thus reducing the system cost and mitigating security risks related to the rare metals.

Many studies have proposed ways to avoid the aggregation of metal particles on a support,^9^ such as the development of a series of “super intelligent” catalysts,^10^ enhanced metal–support interactions,^11–13^ and coating metal nanoparticles with thin metal oxide layers.^14–20^ However, the aggregation of the supported Pd nanoparticles under high-temperature treatment is complicated, and redispersion and sintering can occur during calcination in the presence of oxygen.^21–23^ Two-dimensional surface Pd oxides during oxidation have been considered to be responsible for the redispersion of the Pd nanoparticles. Because the redispersion temperature depends on the Pd particle size, there is a very complex trade-off between the redispersion and aggregation of Pd nanoparticles. Such complex physicochemical phenomena lead us to fabricate an ensured protection layer on nanoparticles by encapsulation.^24^

Indeed, while there are many reports on the encapsulation of metal nanoparticles by metal oxide protection layers, most of them demonstrated self-assembly of metal alkoxides on colloidal metal nanoparticles.^25–31^ The assembly of metal alkoxide precursors with a structure directing agent (SDA) can be induced by surfactant molecules or a microemulsion environment around the metal nanoparticles, resulting in the formation of gas-permeable metal oxide layers. Although these synthetic methods are well established in terms of materials chemistry, they are not suitable for industrial catalytic applications that require robust supports. Nevertheless, there are very limited examples of fabricating metal oxide protective layers on supported metal nanoparticle catalysts. Stair *et al.* developed a metal oxide decoration method on supported metal catalysts using atomic layer deposition (ALD).^32,33^ Appropriate thermal treatment of the encapsulated catalyst can induce gas permeability of the metal oxide layers by the formation of cracks. However, large-scale manufacturing processes using ALD have not yet been established due to the requirement of vacuum conditions and extraordinary devices. Although strong metal–support interaction (SMSI) under reducing conditions that can also induce spontaneous encapsulation of supported metal nanoparticles is well studied,^34,35^ the SMSI state is not stable during the oxidative exhaust-gas purification reaction.

In this study, we demonstrated a photocatalytic fabrication method of an ultrathin SiO_2_ layer selectively decorated on already supported Pd metal nanoparticles to develop a sintering-resistant exhaust gas purification catalyst. The SiO_2_ layer decoration was done using a slurry consisting of Pd/TiO_2_ catalysts, tetraethylorthosilicate (TEOS) as a silica source, aqueous NaNO_3_ as an electrolyte, and the tetramethylammonium cation (TMA^+^) as an SDA. Thermal decomposition of the SDA endows the SiO_2_ layer with gas permeability, which enables the reactant exhaust gas components to get access to the Pd surface. This fabrication method is an application of our previous report on the decoration of a SiO_2_ layer over the Pt/SrTiO_3_ photocatalyst to develop efficient particulate photocatalysts for overall water splitting.^36^ The role of the SiO_2_ shell on Pt/SrTiO_3_ is to function as a nanomembrane; H_2_ generated inside the shell should escape through it, but O_2_ generated outside cannot reach the Pt surface, preventing the reverse reaction of the overall water splitting. This reaction proceeded at close to room temperature in the liquid phase. The current paper investigates whether this nanomembrane selectively decorated on the supported metal particles can be applied to high-temperature exhaust gas purification. The physicochemical role of the SiO_2_ layer in sintering-resistance of supported Pd nanoparticles was investigated. To apply the fabrication method of the SiO_2_ layer for universal support materials, which are not specifically designed as a photocatalyst, precise control of the pH of the SiO_2_ deposition solution is very crucial for effective SiO_2_ decoration on Pd nanoparticles.

## Experimental

### Materials

All chemicals were used as received. Rutile TiO_2_ (<5 μm, ≥99.9%), tetramethylammonium bromide (TMAB, ≥98.0%), NaNO_3_ (≥99.0%), sodium hydroxide (NaOH, 99.9%), hydrochloric acid (HCl, 37%), methanol (99.5%), and ethanol (99.5%) were purchased from Sigma--Aldrich. HNO_3_ solution containing Pd(NO_3_)_2_ (0.216 M) was purchased from Kojima Chemicals Co., Ltd. Tetraethyl orthosilicate (TEOS, >97.0%) was purchased from Tokyo Chemical Industry.

### Preparation of Pd-impregnated TiO_2_

The Pd/TiO_2_ catalyst was prepared by a simple impregnation method. TiO_2_ rutile (0.950 g) was dispersed in a mixture of ultrapure water (20 mL) and Pd(NO_3_)_2_·HNO_3_ solution (0.202 or 1.01 mL corresponding to 1 or 5 wt% Pd). The resulting slurry was ultrasonicated for 15 min and stirred while heating in a water bath at 100 °C until the water completely evaporated. Next, the sample was placed in a muffle furnace and calcined under static air for 2 h at 500 °C, 700 °C, or 900 °C with a heating rate of 5 °C min^−1^. Finally, the products were reduced under pure H_2_ for 2 h at 150 °C with a heating rate of 5 °C min^−1^.^36–38^ The obtained catalysts were denoted as Pd/TiO_2_-500, Pd/TiO_2_-700, and Pd/TiO_2_-900, respectively.

### Photocatalytic performance test of Pd/TiO_2_

A photocatalytic performance test was conducted on the 5 wt% Pd/TiO_2_ catalyst to see whether the excited electrons from TiO_2_ could be utilized in this reaction. The Pd/TiO_2_ catalyst (0.010 g) was dispersed in 10 mL of an aqueous solution of 10 vol% MeOH. The slurry was then irradiated with UV light under ambient pressure with stirring. The amount of evolved H_2_ gas was quantified using an online GC (GC-8A, Shimadzu) equipped with a thermal conductivity detector (TCD). The apparent quantum yield (AQY) of the catalyst was calculated using the following equation:
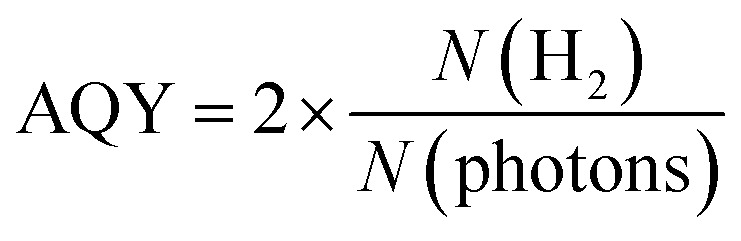
where *N*(H_2_) and *N*(photons) denote the number of H_2_ molecules produced and the number of photons reaching the surface of the reaction solution, respectively.

### SiO_2_ precipitation by increasing pH

A solution was prepared for the SiO_2_ precipitation test by dissolving TMAB (5.25 g) and NaNO_3_ (0.852 g) in a mixture of ultrapure water (100 mL) and ethanol (100 mL). TEOS (15.2 mL) was then added into the solution after the pH was adjusted to 3 with HCl (1.0 M). After 2 h of stirring, NaOH aqueous solution (10 mM) was added drop by drop to increase the pH. With every increase in pH of 0.5, the UV-Vis absorption spectrum and dynamic light scattering (DLS) of the solution were measured using a JASCO UV-Vis V-770 (wavelength range of 200 nm to 2700 nm with a scan speed of 400 nm min^−1^) and HORIBA nanoPartica SZ-100, respectively. For the DLS measurement, the diameter of the precipitates was determined from the average of 10 measurements.

### Preparation of the Pd@SiO_2_/TiO_2_ structure

SiO_2_ layer deposition on Pd/TiO_2_ was performed in a solution consisting of TMAB, NaNO_3_, and TEOS. TMAB (7.86 g) and NaNO_3_ (1.28 g) were dissolved in a mixture of ultrapure water (150 mL) and ethanol (150 mL), and then TEOS (22.8 mL) was added to the solution after the pH was adjusted to 3 with HCl (1.0 M). After 2 h of stirring, Pd/TiO_2_-500 (0.150 g) was dispersed in the solution, and the pH was adjusted to 5 by the addition of an aqueous solution of NaOH (0.10 M). The resulting slurry was irradiated with UV light (Asahi Spectra Co., Ltd., CL-H1-365-9-1) for 0.5 h with stirring and Ar bubbling. The UV light had a peak wavelength of 370 nm and a power of 870 mW.

The precipitates were separated by centrifugation and dried for 16 h at 110 °C with a heating rate of 5 °C min^−1^. They were then calcined under static air for 2 h at 500 °C, 700 °C, or 900 °C with a heating rate of 5 °C min^−1^ in a muffle furnace. Finally, the catalysts were reduced under 30 mL min^−1^ of pure H_2_ for 2 h at 150 °C with a heating rate of 5 °C min^−1^. They were denoted as Pd@SiO_2_/TiO_2_-500, Pd@SiO_2_/TiO_2_-700, or Pd@SiO_2_/TiO_2_-900, respectively.

### Carbon monoxide chemisorption

The obtained catalysts (0.040 g) were packed in a U-shape quartz tube and pretreated under 4% H_2_ balanced with Ar (10 mL min^−1^) at 150 °C for 30 min with a heating rate of 40 °C min^−1^. After purging with pure Ar (10 mL min^−1^) at 150 °C for 30 min, the catalysts were cooled down to 10 °C with ice packs. Subsequently, 500 μL of diluted CO gas (Ar balance, 0.4%) was injected every 8 min until CO was no longer adsorbed. The amount of CO that slipped through without being adsorbed was quantified using a gas chromatograph with a flame ionization detector (SHIMADZU GC-FID, GC-8A) and a methanizer (SHIMADZU MTN-1) and calculated from the peak area.

### Characterization

Transmission electron microscope (TEM) images were obtained using a JEOL 2000EX-II (acceleration voltage: 200 kV) equipped with a CCD camera. The specimens for TEM were prepared by placing drops of the sample dispersed in EtOH onto a 200-mesh copper grid coated with an amorphous carbon film with holes. Scanning transmission electron microscope images and energy dispersive spectroscopy mappings (STEM/EDS) were obtained using an FEI Talos F200X equipped with a Super-X (FEI) and SDD detector (Bruker). Nitrogen adsorption–desorption isotherms were recorded using a MicrotracBEL BELMETAL III. Prior to the measurement, the samples were dried under vacuum at 100 °C for 2 h.

Elemental analysis of the catalysts was performed using a scanning electron microscope and an energy dispersive X-ray spectrometer (SEM/EDX, Hitachi High-Tech S-4700-D). The length of the measurement was 120 s, and the acceleration voltage was 20.00 kV. Inductively coupled plasma optical emission spectrometry measurements were also performed (ICP-OES, Agilent 5110 VDV). X-ray photoelectron spectroscopy (XPS) was performed using a JEOL JPS-9010 MC with a Mg Kα X-ray source. The powder sample was deposited on a metal nickel plate, and powder X-ray diffraction (XRD) patterns of the catalysts were collected on a D/texUltra UltimaIII operated at 40 kV and 40 mA. The samples were scanned from 20° to 60° at 10° min^−1^, and the average of 10 measurements was taken.

### Catalytic performance test

Catalytic reaction rates were measured using a fixed bed catalyst reactor system (MicrotracBEL, BELREA). First, 0.100 g of each catalyst was pretreated at 500 °C in both O_2_ flow and H_2_ flow for 15 min each. The catalytic performance was then tested in 400 mL min^−1^ of simulated exhaust gas, which consisted of 500 ppm of NO, 5000 ppm of CO, 400 ppm of C_3_H_6_, 10% H_2_O, 14% CO_2_, 4900 ppm of O_2_, and 1700 ppm of H_2_ in N_2_ balance and ambient pressure. The reaction temperature was 200 °C to 500 °C. The outlet gas was analyzed by FT-IR (Best Instruments Co., Ltd., Bex-1000FT), an oxygen meter, and a hydrogen meter (Best Instruments Co., Ltd., Bex-520M).

## Results and discussion

### Photocatalytic shell formation scheme

**Fig. 1:**
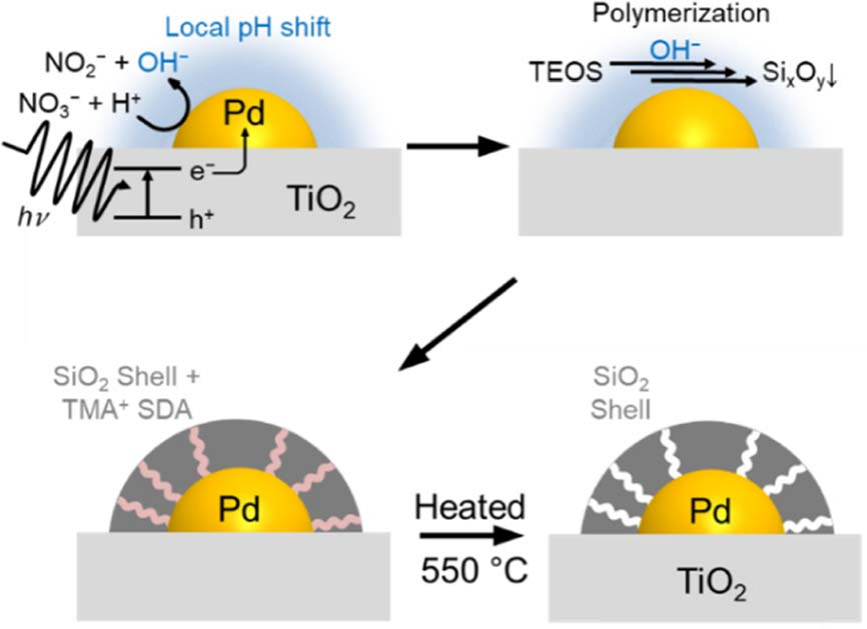
Scheme of SiO_2_ shell formation on supported Pd nanoparticles under UV light irradiation.

Fig. 1 illustrates the shell formation scheme. Under UV light irradiation, the electrons generated by the photoexcitation of TiO_2_ can be transferred to Pd nanoparticles. Next, the excited electrons in the Pd nanoparticles produce H_2_ or reduce NO_3_^−^ as in the following equations, resulting in higher local pH in the vicinity of the Pd nanoparticles.

1 2H^+^ + 2e^−^ → H_2_

2 NO_3_^−^ + H^+^ + 2e^−^ → NO_2_^−^ + OH^−^

The locally increased pH induces the polymerization reaction of TEOS to form Si_*x*_O_*y*_ oligomers, which are deposited on the Pd surface with TMA^+^ by electrostatic force.^39–41^ Finally, the removal of TMA^+^ by the calcination process forms micropores in the SiO_2_ shell, which allows gas diffusion to the core Pd.^42,43^

### Optimization of SiO_2_ decoration conditions

The photocatalytic activity of 5 wt% Pd/TiO_2_ is shown in Fig. S1,† for which the H_2_ production rate was recorded in the presence of methanol as an electron donor. The 5 wt% Pd/TiO_2_ catalyst (∼0.6%) had an apparent quantum yield (AQY) at 370 nm that was approximately one-hundredth lower than the reported AQY value for SrTiO_3_-based photocatalysts.^39^ Hence, the local pH shift induced by the photocatalysis of 5 wt% Pd/TiO_2_ was expected to be smaller than that of Pt/SrTiO_3_. Therefore, prior to the preparation of the catalyst, the threshold pH for SiO_2_ precipitation was precisely measured. Based on the results, the initial pH for SiO_2_ decoration on Pd/TiO_2_ was determined to be slightly lower than the threshold pH.

**Fig. 2:**
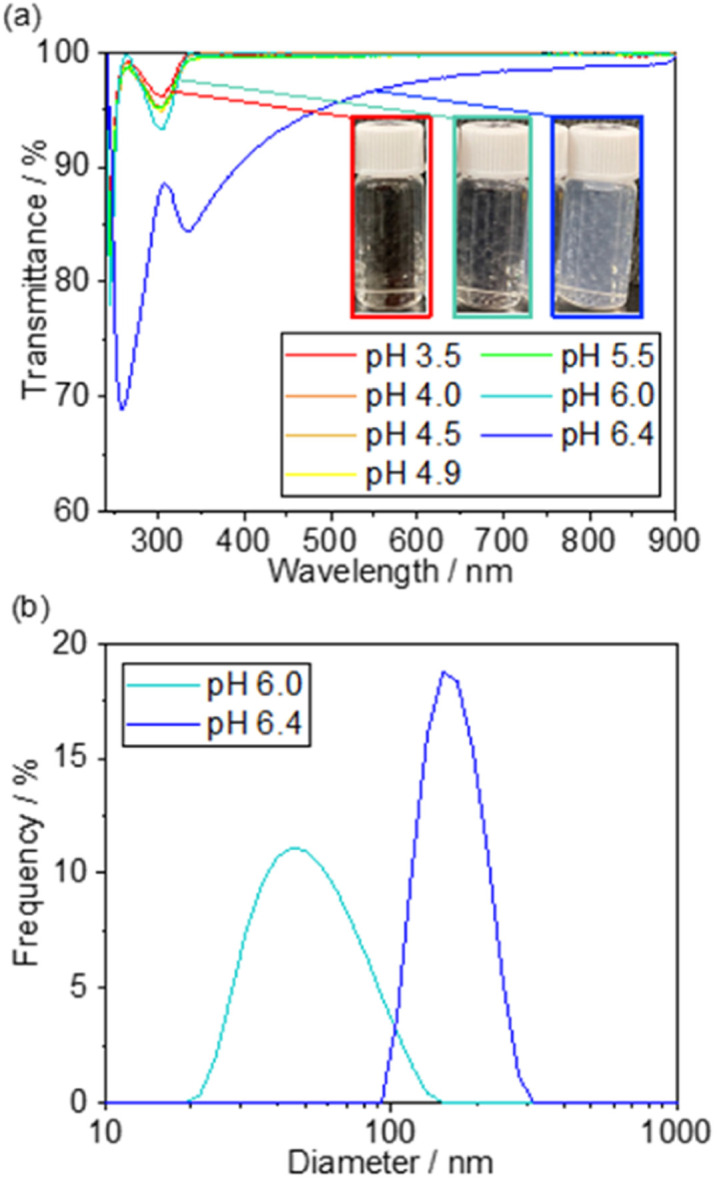
SiO_2_ precipitation test results. (a) UV-Vis transmission spectra of the solutions consisting of TEOS, NaNO_3_, and TMAB at various pH controlled by the addition of aqueous NaOH solution. (b) The particle size distribution of the precipitates in the solution at pH 6.0 and 6.4 obtained by DLS measurement.

Fig. 2a shows the UV-Vis transmission spectra of the solutions consisting of TEOS, NaNO_3_, and TMAB at various pH controlled by the addition of aqueous NaOH solution. The transmittance is hardly changed in the range of pH 3.5–5.5, but it begins to decrease slightly at pH 6.0 and significantly drops at pH 6.4. These results demonstrate that a small amount of Si_*x*_O_*y*_ nanoparticles were formed at pH 6.0, and more were produced at pH 6.4. The formation of the nanoparticles was also observed by DLS measurements, as shown in Fig. 2b.

Below pH 6, almost no particles were present in the solution, which is consistent with the non-reproducible histograms derived from small fluctuations of the laser. Particles with a size distribution of 20–100 nm were observed at pH 6.0, which increased to 100–300 nm at pH 6.4. These results suggest that the formation of Si_*x*_O_*y*_ precipitates by dehydration condensation of TEOS-derived Si(OH)_4_ began at pH 5.5–6.0. Based on the results, the initial pH of the solution for SiO_2_ deposition was set to 5.

### SiO_2_ shell decoration on Pd/TiO_2_

**Fig. 3:**
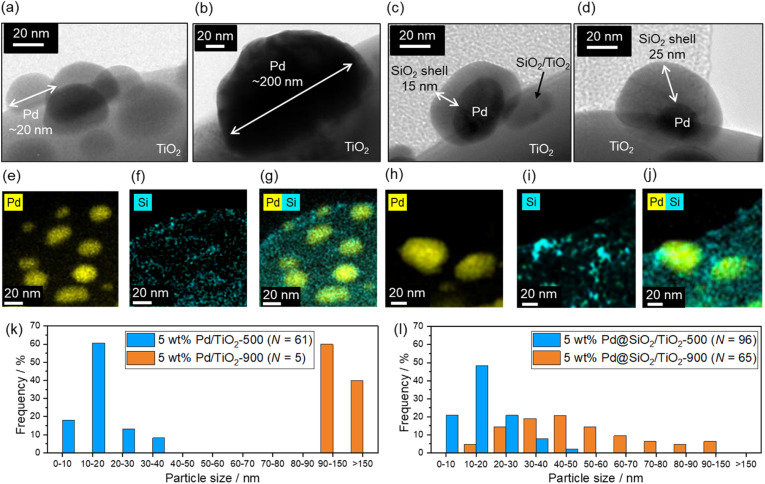
TEM images of (a) 5 wt% Pd/TiO_2_-500, (b) 5 wt% Pd/TiO_2_-900, (c) 5 wt% Pd@SiO_2_/TiO_2_-500, and (d) 5 wt% Pd@SiO_2_/TiO_2_-900, respectively. STEM/EDS mapping images of (e–g) 5 wt% Pd@SiO_2_/TiO_2_-500 and (h–j) 5 wt% Pd@SiO_2_/TiO_2_-900. Histogram of Pd particle size on (k) 5 wt% Pd/TiO_2_-500 and 5 wt% Pd/TiO_2_-900 obtained from TEM images, and (l) 5 wt% Pd@SiO_2_/TiO_2_-500 and 5 wt% Pd@SiO_2_/TiO_2_-900 obtained from STEM/EDS mappings.

Fig. 3 shows the TEM images of the 5 wt% Pd/TiO_2_-500, 5 wt% Pd/TiO_2_-900, 5 wt% Pd@SiO_2_/TiO_2_-500, and 5 wt% Pd@SiO_2_/TiO_2_-900 catalysts. Pd nanoparticles with a size of about 5–30 nm are observed in 5 wt% Pd/TiO_2_-500 (Fig. 3a), but after 900 °C heat treatment in air, the Pd nanoparticles are sintered and have a particle size of more than 100 nm air (5 wt% Pd/TiO_2_-900, Fig. 3b). Histograms of Pd particle size on 5 wt% Pd/TiO_2_-500 and 5 wt% Pd/TiO_2_-900 obtained from TEM images are shown in Fig. 3k. The mean particle size increased from 16 nm to over 130 nm. The distribution of Pd particle size was statistically enlarged due to sintering induced by high-temperature heat treatment. The TEM image of 5 wt% Pd@SiO_2_/TiO_2_-500 in Fig. 3c shows that Pd nanoparticles with a size of ∼40 nm are covered by an amorphous shell with a thickness of ∼15 nm. Other TEM images of 5 wt% Pd@SiO_2_/TiO_2_-500 are shown in Fig. S2.† The particle size of Pd nanoparticles was roughly ∼20 nm and the thickness of the SiO_2_ layer was ∼20 nm.

To confirm that the amorphous shell consists of SiO_2_, STEM/EDS mapping images of Pd and Si distribution on the catalysts were acquired (Fig. 3e–g). The distribution of Si is localized around the Pd nanoparticles in both 5 wt% Pd@SiO_2_/TiO_2_-500 and 5 wt% Pd@SiO_2_/TiO_2_-900, demonstrating that the amorphous shell layer on Pd nanoparticles in Fig. 3c should be SiO_2_. Similar to the 5 wt% Pd/TiO_2_, SiO_2_ decoration on 1 wt% Pd/TiO_2_ was also demonstrated, as shown in Fig. S3.† The Pd nanoparticles with a size of about 5 nm were covered by amorphous SiO_2_ shells with a thickness of a few nanometers. Fig. S4† shows the SEM image of 5 wt% Pd@SiO_2_/TiO_2_-500. The particle size of the TiO_2_ was ∼1 μm.

To investigate the importance of UV light irradiation for uniform decoration of the SiO_2_ shell on Pd nanoparticles, TEOS treatment of Pd/TiO_2_ catalysts under dark conditions was performed as a control experiment. The TEM image of the Pd/TiO_2_ after TEOS treatment under dark conditions (Fig. S5†) shows sparse deposition of amorphous SiO_2_ clusters on the entire Pd/TiO_2_ surface. Unlike Pd@SiO_2_/TiO_2_ catalysts prepared by UV-light irradiation, the Pd nanoparticles were not covered by the uniformly decorated SiO_2_ nanoparticles. Therefore, the photocatalytic reaction induced by UV light irradiation is essential for the uniform SiO_2_ shell decoration on Pd nanoparticles.

Weight fractions of Si and Pd in 5 wt% Pd@SiO_2_/TiO_2_-500 were determined by ICP-OES measurement and the results are shown in Table 1. The weight percent of Pd was measured to be 5.0 wt%, which is equal to the Pd quantity in the impregnation solution. The weight fraction of SiO_2_ was determined to be 5.9 wt%. No impurities of Na and Br were detected. According to the density of the Pd and SiO_2_ (12.0 g cm^−1^ and 2.65 g cm^−1^, respectively), the volume ratio between the Pd and SiO_2_ was calculated as 1:5.3.

**Table 1:** ICP results of 5 wt% Pd@SiO_2_/TiO_2_-500 and 5 wt% Pd@SiO_2_/TiO_2_-900

Sample	Component ratio/wt%			Si/Pd ratio
	Pd	SiO_2_	TiO_2_	
5 wt% Pd@SiO_2_/TiO_2_-500	5.0	5.9	89	2.1
5 wt% Pd@SiO_2_/TiO_2_-900	5.1	6.3	89	2.2

Assuming that all the SiO_2_ forms a hemispherical shell on the hemispherical Pd nanoparticles, the thickness of the shell should be about 0.4 times the diameter of the Pd nanoparticles. Hence, if the Pd grain size is 40 nm, the thickness of the SiO_2_ shell is calculated to be 16 nm, which is roughly consistent with the TEM result (Fig. 3c). Therefore, the weight content of the SiO_2_ (∼6 wt%) should be reasonable. SiO_2_ particles were also observed on the TiO_2_ surface because SiO_2_ precursors are unavoidably adsorbed on the TiO_2_ surface.

The Pd particle size is almost maintained even after heat treatment at 900 °C in air (5 wt% Pd@SiO_2_/TiO_2_-900, Fig. 3d). The diameter of the Pd nanoparticles seems to be slightly increased after heat treatment at 900 °C (Fig. 3h) in comparison to the STEM/EDS mapping images of Pd in 5 wt% Pd@SiO_2_/TiO_2_-500 (Fig. 3e). Histograms of Pd particle size on 5 wt% Pd@SiO_2_/TiO_2_-500 and 5 wt% Pd@SiO_2_/TiO_2_-900 were obtained from the STEM/EDS mapping and shown in Fig. 3l. Although the mean particle size increased from 23 nm to 53 nm after 900 °C heat treatment, the Pd particle size of 5 wt% Pd@SiO_2_/TiO_2_-900 was clearly statistically smaller compared to 5 wt% Pd/TiO_2_-900. The localization of the Si distribution around Pd particles was also measured (Fig. 3i and j), and the weight percent of Pd and SiO_2_ was not changed after heat treatment at 900 °C in air (Table 1). This suggests that the SiO_2_ shell prevents aggregation of the Pd nanoparticles.

Table 2 summarizes the amount of CO adsorbed on 5 wt% Pd/TiO_2_ and 5 wt% Pd@SiO_2_/TiO_2_ after heat treatment in air at various temperatures. The amount of CO adsorbed on 5 wt% Pd/TiO_2_ after heat treatment at 500 °C drastically dropped from 15 μmol g_cat_^−1^ to 2.8 μmol g_cat_^−1^ after heat treatment at 900 °C. The rate of the decrease of the surface Pd site after heat treatment at 900 °C in air was calculated to be 22%. The calculated particle diameter with an assumption of hemispherical Pd particles was 25 nm after heat treatment at 500 °C and 139 nm after heat treatment at 900 °C, which is consistent with the TEM results.

**Table 2:** Results of CO chemisorption capacity (unit: μmol g_cat_^−1^)

Sample	Heat treatment temperature			Rate of decrease by 900 °C heating
	500 °C	700 °C	900 °C	
5 wt% Pd/TiO_2_	15	13	2.8	19%
5 wt% Pd@SiO_2_/TiO_2_	5.0	5.2	4.7	94%
1 wt% Pd/TiO_2_	5.4	n.t.^a^	1.2	22%
1 wt% Pd@SiO_2_/TiO_2_	1.7	n.t.^a^	1.7	100%

a Not tested.

The CO adsorption capacity on 5 wt% Pd@SiO_2_/TiO_2_ was 94% retained even after heat treatment at 900 °C, suggesting that the SiO_2_ decoration suppressed the aggregation of Pd nanoparticles and achieved excellent high-temperature stability. Although the CO adsorption amount decreases for 5 wt% Pd@SiO_2_/TiO_2_-500 (5.0 μmol g_cat_^−1^) compared to 5 wt% Pd/TiO_2_-500 (15 μmol g_cat_^−1^), this result may not simply be due to the SiO_2_ layer inhibiting the adsorption of CO onto the Pd surface. As will be discussed later, the catalytic activity of exhaust gas purification reactions is almost unchanged with or without the SiO_2_ layer. Hence, it can be concluded that SiO_2_ does not block the surface sites, and the decrease in CO adsorption by the SiO_2_ layer modification can be attributed to the difference in the interaction between CO and the Pd surface.

A similar trend was observed when the loaded Pd amount was decreased to 1 wt%. The amount of CO adsorbed on 1 wt% Pd/TiO_2_ significantly drops to 22% after heat treatment at 900 °C in air, but the CO adsorption amount on 1 wt% Pd@SiO_2_/TiO_2_ is well maintained (∼100%) after the heat treatment. Therefore, aggregation of Pd nanoparticles at elevated temperature can be effectively prevented by the shell.

**Fig. 4:**
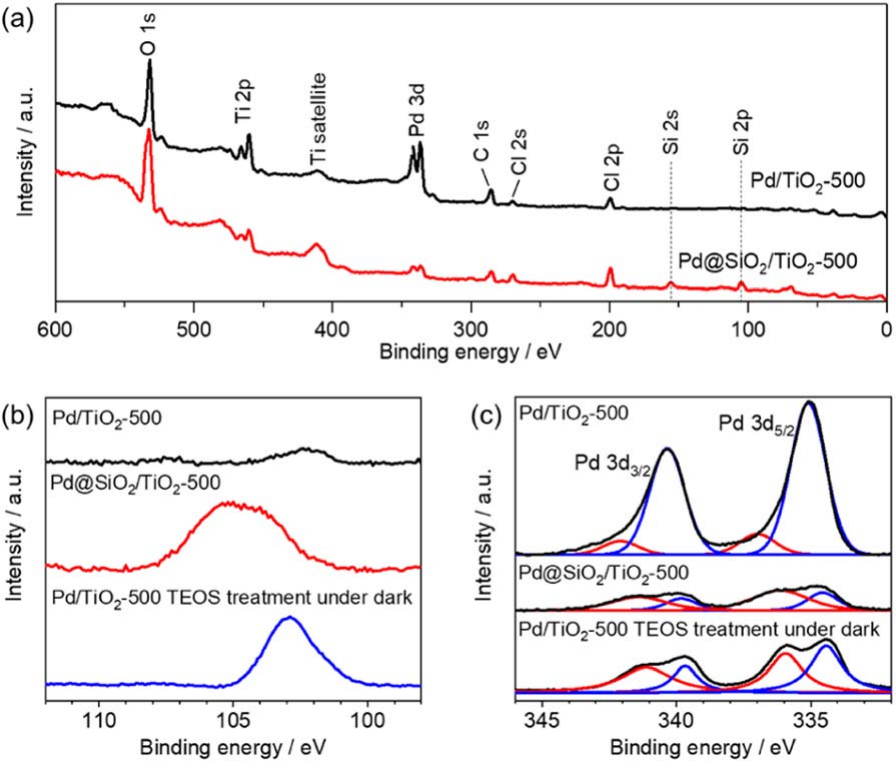
XPS results of 5 wt% Pd/TiO_2_-500 and 5 wt% Pd@SiO_2_/TiO_2_-500. (a) Survey scan, (b) Si 2p region and (c) Pd 3d region.

X-ray photoemission spectroscopy was also performed on 5 wt% Pd/TiO_2_-500 and 5 wt% Pd@SiO_2_/TiO_2_-500. In the survey scan (Fig. 4a), Si peaks that cannot be seen for 5 wt% Pd/SiO_2_/TiO_2_-500 clearly appear for 5 wt% Pd@TiO_2_-500, indicating that SiO_2_ was deposited on the catalyst. The detailed chemical state of Si species on the Pd/TiO_2_ catalyst was characterized using the Si 2p region X-ray photoemission spectrum (Fig. 4b). Based on the binding energy of the Si 2p peaks, the deposited Si species on both Pd@SiO_2_/TiO_2_ and Pd/TiO_2_ after TEOS treatment under dark conditions can be identified as SiO_2_. The spectra show that the Si 2p peak position of Pd@SiO_2_/TiO_2_ was positively shifted compared to the Si 2p peak of the Pd/TiO_2_ catalyst after TEOS treatment under dark conditions. Unlike the SiO_2_ clusters sparsely deposited on the Pd/TiO_2_ catalyst, the SiO_2_ shell localized on the Pd nanoparticles became positively charged probably due to electron withdrawal by Pd nanoparticles.

Fig. 4c shows the detailed spectra of the region of Pd 3d peaks. The peak area decreased after the SiO_2_ decoration, suggesting that the Pd nanoparticles were successfully covered by SiO_2_. Pd 3d peaks were also slightly decreased by the TEOS treatment, suggesting that the Pd nanoparticles should be partially coated with the SiO_2_ clusters. However, the peak intensity is much higher than that of the Pd@SiO_2_/TiO_2_ catalysts prepared by UV light irradiation. Therefore, the photocatalytic reaction triggers uniform SiO_2_ shell formation on Pd nanoparticles, which corresponds to the TEM observation.

Deconvolution revealed the components of metallic Pd (340.5 eV and 335.3 eV) and PdO (342.3 eV and 337.0 eV) in the spectra of both 5 wt% Pd/TiO_2_-500 and 5 wt% Pd@SiO_2_/TiO_2_-500. The ratio of the PdO peak to the Pd metal peak is higher for 5 wt% Pd@SiO_2_/TiO_2_-500, which is probably due to the selective SiO_2_ decoration on metallic Pd or oxidation of the surface Pd species resulting from the formation of an interface with the SiO_2_ shell.

**Fig. 5:**
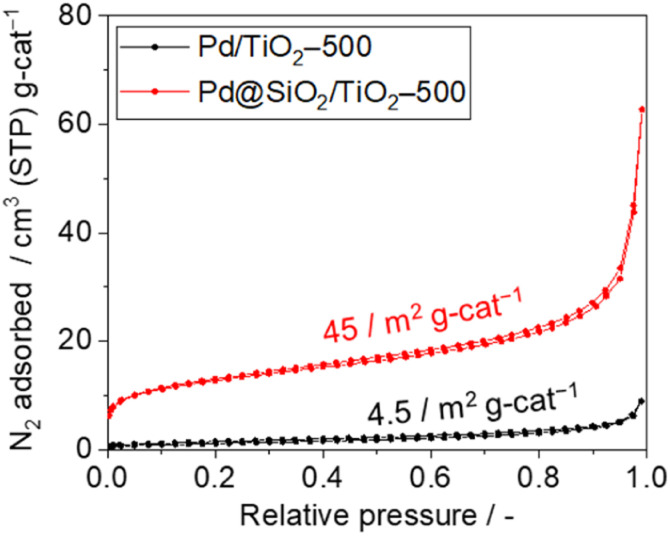
Nitrogen adsorption–desorption isotherms of 5 wt% Pd/TiO_2_-500 and 5 wt% Pd@SiO_2_/TiO_2_-500.

The surface area and porosity of the SiO_2_ shell were examined using nitrogen adsorption–desorption isotherms and carbon monoxide pulsed adsorption measurement, as shown in Fig. 5. The isotherm of 5 wt% Pd/TiO_2_-500 exhibits type II features and a Brunauer–Emmett–Teller (BET) surface area of 4.5 m^2^ g_cat_^−1^ (Fig. 5). However, the isotherm of 5 wt% Pd@SiO_2_/TiO_2_ shows a steep rise in the lower relative pressure region, suggesting the presence of micropores. The BET surface area of Pd@SiO_2_/TiO_2_ was 45 m^2^ g_cat_^−1^. The 10-fold increase in the surface area of 5 wt% Pd@SiO_2_/TiO_2_ compared to Pd/TiO_2_ is attributed to the porous nature of the SiO_2_ shell on Pd.

**Fig. 6:**
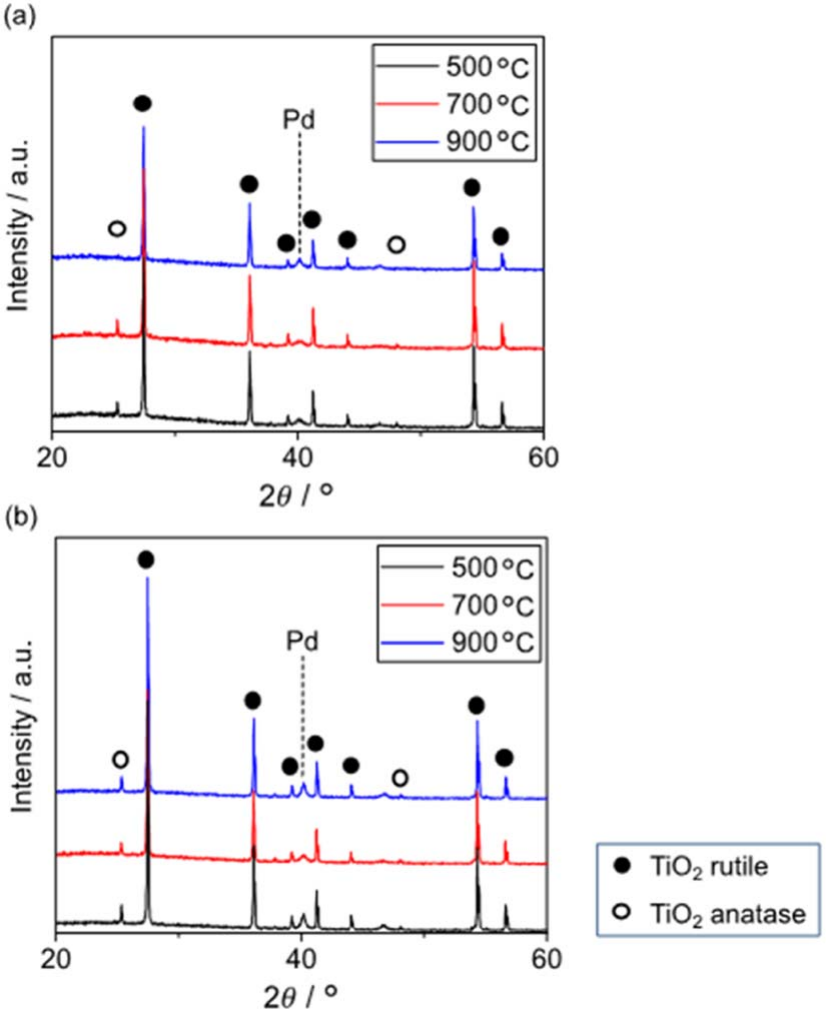
XRD patterns of (a) 5 wt% Pd/TiO_2_-500, 700, 900 and (b) 5 wt% Pd@SiO_2_/TiO_2_-500, 700, 900.

Fig. 6 shows the XRD patterns of 5 wt% Pd/TiO_2_ and 5 wt% Pd@SiO_2_/TiO_2_ after heating at various temperatures. All patterns exhibited a small peak around 40.1° corresponding to Pd^0^(111). The pattern of 5 wt% Pd/TiO_2_ demonstrated that the TiO_2_ support consisted of a major rutile phase and a minor anatase phase. The minor anatase peaks of 5 wt% Pd/TiO_2_ disappeared after heat treatment at 900 °C. Typically, the transition temperature of TiO_2_ without any modifications is around 800 °C. However, 5 wt% Pd@SiO_2_/TiO_2_ showed the anatase peaks even after heat treatment at 900 °C, suggesting that SiO_2_ deposition on the Pd/TiO_2_ shifted the anatase-to-rutile transition temperature of the TiO_2_ support to a higher temperature.

Okada *et al.* reported an upper shift of the transition temperature of TiO_2_ by a few atomic percent with Si doping. This was due to the suppression of surface nucleation sites for rutile by the formation of an amorphous SiO_2_ surface layer.^44^ Similar to this report, the SiO_2_ partially covering the Pd/TiO_2_ applied *via* photodeposition seemed to have the ability to suppress the nucleation sites of rutile TiO_2_.

### Catalytic performance of Pd@SiO_2_/TiO_2_

**Fig. 7:**
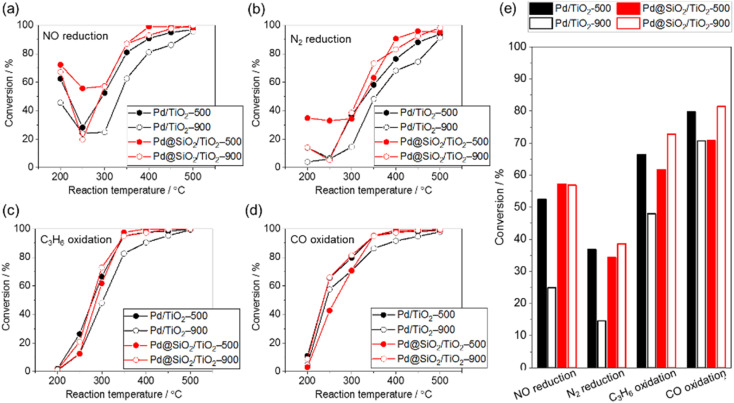
Catalytic performance in simulated exhaust gas (500 ppm NO, 5000 ppm CO, 400 ppm C_3_H_6_, 10% H_2_O, 14% CO_2_, 4900 ppm O_2_, and 1700 ppm H_2_ in N_2_ balance, 400 mL min^−1^, ambient pressure). (a) NO reduction, (b) N_2_ reduction, (c) hydrocarbon oxidation, (d) CO oxidation, and (e) summarized bar graph at 300 °C.

The purification reaction of simulated exhaust gas was examined to investigate the influence of the high-temperature treatment of 5 wt% Pd/TiO_2_ and 5 wt% Pd@SiO_2_/TiO_2_ on the catalytic performance (Fig. 7). The test was carried out under a gas flow of NO (500 ppm), CO (5000 ppm), C_3_H_6_ (400 ppm), H_2_O (10%), CO_2_ (14%), O_2_ (4900 ppm), and H_2_ (1700 ppm) with N_2_ balance at ambient pressure. The conversion of NO reduction, CO oxidation, and C_3_H_6_ oxidation was estimated from the concentrations of NO, CO, and C_3_H_6_ in outlet gas, respectively, while N_2_ reduction was estimated using the NH_3_, NO_2_, and N_2_O concentrations in the outlet gas.

In all reactions, the conversion increased at an elevated reaction temperature, but there was a relatively high conversion of NO and N_2_ reduction at lower temperature due to the selective reduction by H_2_ contained in the reaction gas. In other words, the reducing agent for NO and N_2_ reduction was changed from H_2_ at lower temperature to other gases (*e.g.*, CO or C_3_H_8_) at higher temperature. This occurred because the H_2_ was consumed by the reaction with O_2_ at higher temperature.^45^

The conversion of NO reduction, N_2_ reduction, and C_3_H_8_ oxidation over 5 wt% Pd/TiO_2_ significantly dropped after heat treatment at 900 °C in air due to the aggregation of the Pd nanoparticles. In contrast, Pd@SiO_2_/TiO_2_ maintained the conversion of the NO and N_2_ reduction or improved the conversion of C_3_H_8_ oxidation even after the treatment. This demonstrates the protective role of the SiO_2_ shell on Pd nanoparticles in preventing the thermal drop of the catalytic performance. The catalytic performance of Pd@SiO_2_/TiO_2_-500 for the NO reduction, N_2_ reduction and C_3_H_8_ oxidation was almost corresponding to the performance of Pd/TiO_2_-500. Therefore, the gas permeability of the SiO_2_ shell was sufficiently high not to prevent the catalytic reaction at Pd nanoparticles. Moreover, SiO_2_ decoration does not inhibit the Pd surface site for activation of exhaust gas. On the other hand, the performance of CO oxidation is not significantly changed by the SiO_2_ decoration and probably due to CO diffusion limitation of the CO oxidation kinetics above 277 °C.^46^

There have been no previous demonstrations of the use of these photocatalytically fabricated gas-permeable layers for high-temperature thermal catalytic reactions, and this paper is the first report of such a demonstration. The current photocatalytic fabrication method can be applied for a large variety of gas permeable layers, such as chromium-,^47^ molybdenum-,^48^ titanium-,^49,50^ tantalum-based oxides,^49,50^ and so on. Furthermore, this photo-assisted fabrication method does not require special vacuum conditions and can be applied for mass production using a continuous-flow synthesis system.^51^ This study bridges strategies of photocatalysis-assisted nanostructure synthesis and thermal catalysts with sintering tolerance.

## Conclusions

In this work, ultrathin SiO_2_ shells were successfully synthesized on Pd nanoparticles supported by TiO_2_ by a local pH shift near the supported Pd catalyst using a UV light-induced photocatalytic reaction on the TiO_2_ support. TEM and STEM/EDS mapping images showed that the particle size of the Pd nanoparticles (∼40 nm) with the SiO_2_ shell (∼20 nm) was almost unchanged by high-temperature treatment at 900 °C in air. This suggested that the SiO_2_ shell prevented thermal aggregation of the nanoparticles. The Pd/TiO_2_ without the SiO_2_ shell exhibited a drop in the number of active sites, which was likely due to aggregation of the –Pd catalysts. However, the number of active sites on the Pd@SiO_2_/TiO_2_ catalyst was maintained even after the catalyst was calcined at 900 °C. Consequently, the SiO_2_ shell on Pd nanoparticles prevented the drop in the catalytic performance of the Pd/TiO_2_ catalyst in simulated exhaust gas purification without disturbing the catalytic performance.

## Author contributions

K. T. and H. M. led the research and contributed to the experiment planning, results analysis, and supervision of the research project. A. T. and H. T. carried out the experiments. F. K. and H. T. were involved in the design of experiments, results analysis, and paper writing. All the authors worked on the preparation of the manuscript and provided useful comments.

## Conflicts of interest

There are no conflicts to declare.

## Supplementary Material

NA-005-D2NA00703G-s001
