# Supplementary material for: Photocatalytic formation of a gas permeable layer selectively deposited on supported metal nanoparticles for sintering-resistant thermal catalysis
Source: Nanoscale Adv. 2022 Dec 19;5(4):1124–32. doi: 10.1039/d2na00703g (PMC9926894; doi:10.1039/d2na00703g)
Supplement: NA-005-D2NA00703G-s001 [file NA-005-D2NA00703G-s001.pdf]

Electronic Supplementary Material (ESI) for Nanoscale Advances.
This journal is © The Royal Society of Chemistry 2022

Supplementary information

Photocatalytic formation of gas permeable layer selectively deposited on supported metal nanoparticles for sintering resistant thermal catalysis

*Ayato Takabayashi,[a] Fuminao Kishimoto,[a] Hiroto Tsuchiya,[b] Hitoshi Mikami,[b] Kazuhiro Takanabe,[a]**

[a] Department of Chemical System Engineering, School of Engineering, The University of Tokyo, 7−3−1 Hongo, Bunkyo−ku, Tokyo, 113−8656, Japan

[b] Honda R&D Co., Ltd., 4630 Shimotakanezawa, Haga–machi, Hagagun, Tochigi 321–3393, Japan

KEYWORDS: Core−shell catalyst, Automobile catalyst, Photodeposition, Sintering, Thermal stability

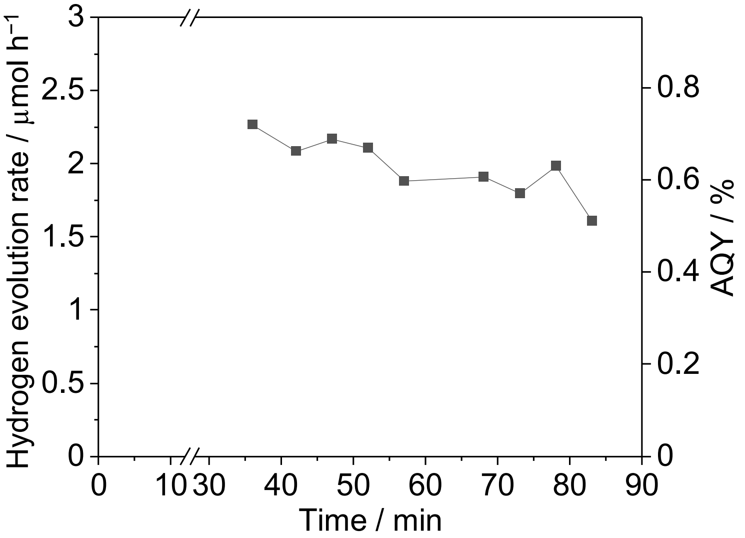

Figure S1. Photocatalytic hydrogen evolution rate on Pd/TiO_2. Pd/TiO_2 (0.010 g) was dispersed in aqueous solution of 10 vol.% MeOH (10 mL), and the slurry was irradiated with UV light (365 nm, 870 mW) under ambient pressure with stirring.

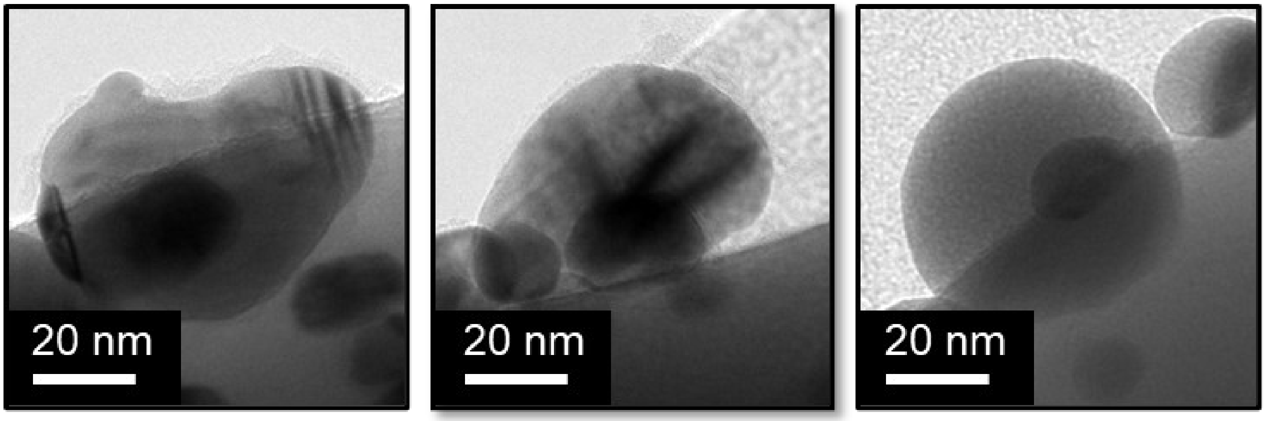

Figure S2. TEM images of 5 wt% Pd@SiO_2/TiO_2–500.

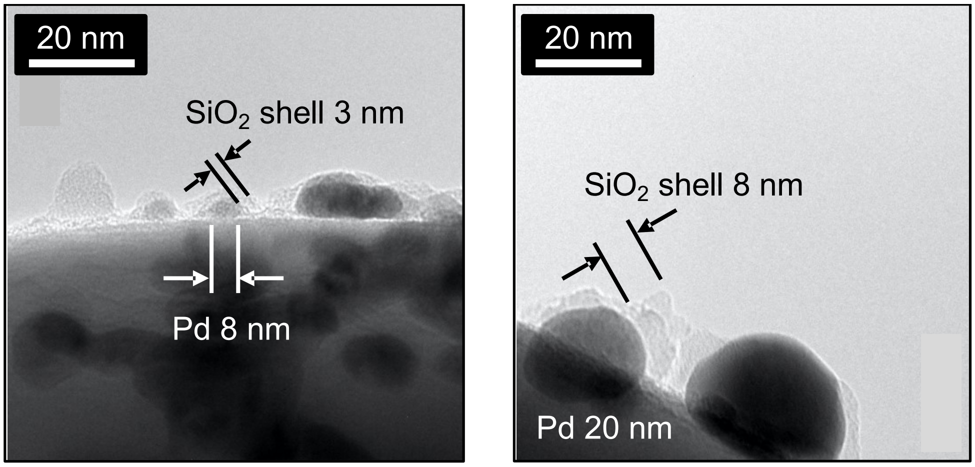

Figure S3. TEM images of 1 wt.% Pd@SiO_2/TiO_2–500. Pd nanoparticles with size of about 5 nm were covered by amorphous SiO_2 shells with a thickness of a few nanometers.

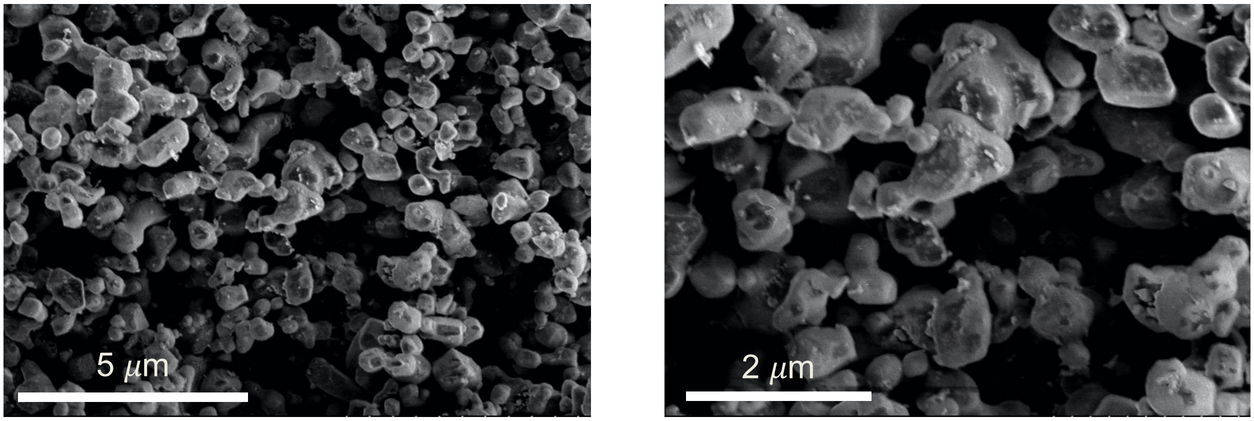

Figure S4. SEM images of 5 wt.% Pd@SiO_2/TiO_2–500.

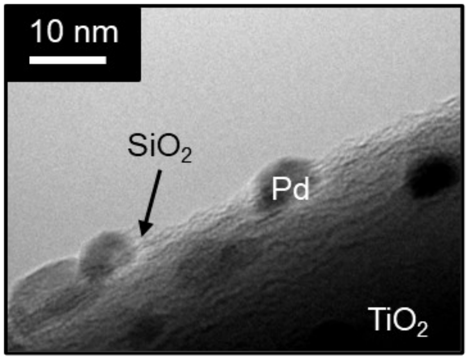

Figure S5. TEM image of the Pd/TiO_2 after TEOS treatment under dark condition.
